# The Oral health status of children with autism Spectrum disorder in KwaZulu-Nata, South Africa

**DOI:** 10.1186/s12903-018-0632-1

**Published:** 2018-10-12

**Authors:** Magandhree Naidoo, Shenuka Singh

**Affiliations:** School of Health Sciences, Discipline of Dentistry, University of KwaZulu-Natal, Westville Campus, South Africa

**Keywords:** Dental caries, Oral health, Plaque, Gingival inflammation, Oral care

## Abstract

**Background:**

Echoing the sentiments of the Sixty-seventh World Health Assembly of May 2014, mandating the all-inclusive and synchronized efforts for the management of autism spectrum disorder (ASD), the aim of this current study was to investigate the oral health status of children with ASD aged between 7 to 14 years in KwaZulu-Natal, South Africa.

**Methods:**

An investigative cross-sectional quantitative design employing non-probability purposeful sampling was conducted on 149 children with ASD attending special needs schools in KwaZulu-Natal. An intra-oral examination to investigate decayed, missing and filled teeth (DMFT/dmft), gingival index (GI), and plaque index (PI), attrition and soft tissue trauma using the World Oral Health Survey Form for Children, (2013) was implemented during data collection.

**Results:**

Average DMFT/dmft scores of 3, 42 and 0, 97 were recorded respectively. Molars dominated the decayed component of the DMFT/dmft with an average caries prevalence of (51, 7% and 40, 8%) respectively. These results displayed zero fillings indicative of unmet treatment needs. The gingival index revealed mild gingival inflammation, (46, 3%) and the plaque index demonstrated visible plaque at (43, 6%).Attrition scores revealed mild loss of dental enamel (47%). The most prevalent soft tissue trauma recorded was lip biting (37, 25%).

**Conclusion:**

Restorative or preventative treatment measures were not evident in this study. Unmet dental needs are therefore an important concern in this population. Health care planners should develop preventive programs targeted at high risk groups such as this study population.

## Background

The South African Government displayed its advocacy for defending and protecting the right to health of all children aged below 18 years by ratifying the United Nations Convention on the Rights of the Child, (1989) [1]. The population with (ASD) presents with similar health problems as that of the typical population, but due to factors including poor dietary preferences; behaviors and specific aversions, this population is at a greater risk and more susceptible to developing chronic non-communicable oral health conditions [2]. The increasing prevalence of ASD coupled with this population being highlighted as one those with the greatest disease burden, has ignited an interest in their oral health concerns, which coincides with one of the global oral health goals to promote oral health within this population group [3]. ASD is a neurodevelopment disorder which presents within the first 3 years of life [4]. Typical manifestation include impaired social interactions; deficits in language acquisition; and restricted and repetitive behaviours [5]. The World Health Organisation has estimated the global prevalence of ASD to be 1:160 persons [6]. This is an average figure that however, does not include the prevalence of this condition in Sub-Saharan Africa. The scarcity of this data has been attributed to the rarity of epidemiological surveys on ASD in this geographical area [7]. Some data extracted predominantly from studies conducted at hospitals such as in South-West Nigeria documented a prevalence rate of 1:43.5; South-Eastern Nigeria, revealed a prevalence rate of 1:125; and Uganda, documented a prevalence of 6.8:1000 [8, 9]_._Unfortunately due to the absence of epidemiological studies in South Africa, accurate national autism statistics in South Africa are not available. Global prevalence rates according to The World Health Organization [6] is currently being used.

There are no typical unusual oral manifestations present in individuals with ASD. However due to problematic behaviors; unusual oral habits; medications; and dietary preferences, this population appears to be at risk for the development of oral diseases [10, 11]. It has been estimated that almost 70% of individual with ASD present with self-injurious behaviours (SIB) located in the head and neck region [12]. Oral findings reported in patients with autism spectrum disorder included traumatic ulcerated lesions frequently a consequence of (SIB) such as head banging; face tapping; and gingival picking [13]. Another consequence of SIB noted in autism is auto extraction. Auto-extraction refers to the self- removal of teeth [14]. Unusual oral habits include bruxism, tongue thrusting, non-nutritive chewing on objects such as gravel, cigarette butts, or pens and repeated regurgitation [15]. The most frequently reported dental implications of bruxism in children with autism spectrum disorder include joint pain in the temporomandibular area; excessive wear of the dental enamel; and tooth avulsion [16]. Attrition has also been a consequence of a condition frequently seen in ASD called Pica. Lithophagia, a type of Pica involving the eating of grit and stones has been documented to lead to attrition and enamel wear [17]. Conditions such as over jets, spacing, Class II molar inclinations and open bites were also reportedly higher in patients with ASD [18].

Children with ASD are often cited as having certain food preferences, which includes sweets and soft foods [19]. Additionally manual dexterity required for adequate tooth brushing is often reduced in children with ASD, resulting in inadequate tooth brushing [2]. Dental care access and oral care delivery has proved to be very problematic for children with ASD. Financial costs, scarcity of medical aid coverage and the child’s indifference towards dental procedures has been acknowledged as the core liabilities to oral care delivery for this population [20].

The maintenance of optimum oral care in children with ASD ought to be of significant focus, since existing oral care problems may intensify, thereby impacting the individual’s overall quality of life [21]. Accordingly, an essentiality for a holistic approach to oral care in children with ASD is required [22]. However conflicting research results globally, on the oral care status of children with ASD has placed oral care interventions in this population at a crossroad [23]. Several studies have reported poor oral hygiene in children with ASD [2, 9, 24–27]_,_ whilst other research reported better oral hygiene in participants with ASD [28–32], and comparable oral health was also recorded for this population [33, 34]. This confliction is inflated by the non-existence South African epidemiological data. Data which is vital to inform and plan preventative oral care measures is unfortunately, to the best of our knowledge, not available. Global oral health studies conducted in this population is therefore considered as a point of reference for the said study. The conflicting status of oral care in children with ASD presents a dilemma. Research is anticipatory to elucidate our understanding of ASD and oral care, specifically the oral care status of this population that will serve to inform future oral care measures and procedures.

The numerous studies conducted abroad on the ASD population have shown that children with autism spectrum disorder have either statistically higher; lower or similar caries experience when compared to those without this condition [2, 9, 24, 28, 29, 33, 34]. South African epidemiologic data on the oral care status of children with ASD is lacking. Therefore the aim of this study was to create an epidemiological oral care profile for children with ASD aged between 7 to 14 years attending special needs schools in KwaZulu-Natal, South Africa, by investigating the oral health status within the parameters of the DMFT/dmft; GI; PI; attrition and soft tissue trauma.

## Methods

### Study design

This study was conducted by means of a cross-sectional design, using quantitative data. In order to establish the burden of disease in a specific population, which can subsequently be used for intervention and health planning programs, data was collected at a specific time and analyzed to evaluate the oral health status of children with autism spectrum disorder.

### Setting of the study

This research was undertaken in KwaZulu-Natal, one of 9 provinces in South Africa. A list from the (KwaZulu-Natal Department of Education) identified 45 special needs schools in this province. Personal communication into these 45 special needs schools revealed that only 20 of these 45 schools cater for children with ASD in KwaZulu-Natal.

### Study population

Children aged between 7 to 14 years, attending identified special needs schools in KwaZulu-Natal, who were diagnosed with autism spectrum disorder according to the DSM-V criteria by a qualified professional were included in this study. The inclusion criteria also stipulated that participants would only be included after parental consent and individual assent was obtained. The exclusion criteria included Children who presented with any other co-existing condition; with no formal diagnosis; children whose parents did not consent to the study and those who did not assent to the study were excluded from the study. This study intended to exclude children who had visited the dentist within the last 3 months of data collection to minimize any potential bias because it was anticipated that any dental intervention could potentially impact on the results of the GI scores. This exclusion was however revised and was not included as an exclusion criteria. The population size for children with ASD in KwaZulu-Natal due to the absence of existing data could not be ascertained. Data collection was subjected to the response rate from the special needs schools. At a response rate of 80, 9%, a total of 149 children with ASD were included in this study.

### Data collection

Data was collected by a single researcher, an oral hygienist registered with the Health professions Council of South Africa (HPCSA), employed within the academic discipline of dentistry. The researcher was calibrated by academics involved in caries research. Calibration was performed according to the standards set out by The British Association for the Study of Community Dentistry (BASCD). During the process of data collection involving a single researcher, it is often questioned if the researcher will record and interpret a finding in the same manner when presented with the same situation twice. Intra-examiner reliability was observed by adhering to the guidelines of the World Health Organization [35] where every 5th participant was re-examined and data recorded on a separate data sheet to be compared later. Data was recorded by a registered dental therapist. The Kappa statistic were employed to ascertain intra-examiner reliability on 29 participants. A kappa value for DMFT (0,915); GI (0,920); PI (0,880); attrition (0,824) and soft tissue trauma (0,930) was reached.

Decayed, missing and filled index (DMFT/dmft); gingival index (GI); plaque index (PI); attrition; and soft tissue trauma was assessed using the world oral health survey, (2013) [36]. The Gingival Index [37] was used to assess the gingival status and to record clinical variations in the gingiva. Due to the nature of the participants, this examination was adapted to evaluate the overall gingival status by means of a visual examination. The bleeding upon probing of selected index teeth was omitted to avoid participant anxiety. Based on the visual examination, changes in color; size and shape were observed and scored. Gingiva observed with no clinical variations in color; size and shape was scored 0 and categorized as normal. Gingiva observed with slight changes in color, size and shape was scored 1 and categorized as mild gingival inflammation. Gingiva observed with changes in color; size and shape accompanied by visible signs of bleeding was scored 2 and categorized as moderate gingival inflammation. Gingiva observed with distinctive gingival enlargement and spontaneous bleeding was scored 3 and categorized as severe gingival inflammation. Similar adaptations were made in a study on the oral health status of children with ASD in Chennai [35]. These adaptive measures were employed in the Chennai study to accommodate the characteristic behaviors of these participants. These authors have agreed that these adaptations did not show any evidence of compromise in the validity of the results.

Plaque accumulation was recorded using the plaque index [38]. Attrition and soft tissue trauma was recorded according to the categories in the (World Oral Health Survey, 2013) [39]. All infection control procedures were maintained according to the Center for Disease Control (CDC) guidelines and waste was disposed of according to the waste removal guidelines of the Health Professions Council of South Africa.

Approval to conduct this research was obtained from The Biomedical Research and Ethics Committee (BREC^1^) at The University of KwaZulu-Natal; the KwaZulu-Natal Department of Education (KZN-DOE^2^); and the KwaZulu- Natal Department of Health (KZN-DOH^3^). Consent for participation was obtained from the participating school principals; teachers; and parents of selected children. ASD has varying degrees of impairment that may impact cooperation during data collection. The designated school therapist assisted with the selection of participants based on the functioning of the child. Higher functioning children were selected by the therapist for participation in this study. Assent was obtained from the participant prior to the commencement of data collection. Provisional dates for the data collection were sent to the participating schools. This assessment was conducted in accordance with the guidelines of the World Oral Health Survey [39]. Each participant was allowed to assent to the study using a picture communication board in the presence of the teacher or teaching assistant. The facilitator/ teacher escorted the consenting participant to the mobile dental unit. A picture communication schedule, prepared using the Boardmaker Software® was used to explain the procedure to both the facilitator/teacher and the participant. The participant was seated on the dental chair. An extra-oral examination to assess the presence of any scars; lip biting; trauma to the head and neck; hands and fingers; and signs of self-injurious behaviors was conducted first. An intra-oral examination of the soft and hard tissues was conducted using disposable gloves, disposable tongue depressors for retraction and sterilize gauze. This examination assessed decayed, missing and filled teeth according to the DMFT/dmft index. Gingival inflammation; plaque accumulation; attrition; and soft tissue trauma were investigated according to categories of the World Oral health Survey, 2013. All disposable medical waste was disposed of in designated containers according to the HPCSA guidelines and returned to the oral and dental training hospital to be collected by an accredited waste removals service provider. The participants were thanked and provided with a sample toothbrush and toothpaste as a token of appreciation and escorted back to the classroom. A research assistant recorded all data.

Data was analysed using IBM SPSS version 24.0. Descriptive statistics included the use of frequency tables, cross-tabulations and means with standard deviations. Inferential statistics used the Fisher’s Exact test to determine relationships between the status of teeth and factors of interest such as (gender; location and age). The Kolmogorov Smirnov test revealed that the data was not normally distributed and means companions were done using the Kruskal Wallis tests. Kappa statistics was used to determine intra-examiner reliability.

## Results

A response rate of 80, 97% (*n* = 149) was recorded. The majority of participants of this study were male, (71, 1%). Most participants indicated that they live in Peri-urban geographical areas, (78%). The age distribution is reflected in Table 1.

**Table 1:** Gender; Age; And Geographical Distribution of Children with ASD in KwaZulu-Natal (*n* = 149)

Gender	n (%)
Female	43 (28,9)
Male	106 (71,1)
Geographic Location	
Suburban	116 (77,9)
Rural	33 (22,1)
Age in Years	
7–8	30 (20,1)
9–10	37 (24,8)
10–11	37 (24,8)
12–14	45 (30,2)

A mean score of 3, 07 for decayed teeth (D); missing teeth (M) was 0, 39; and filled (F) 0, was recorded in the permanent dentition. An average DMFT score was recorded at 3, 42. Average caries prevalence in the permanent molars was recorded at 51, 7%; 12, 4% in the premolars; 3, 7% in the canines and in the incisors. As indicated in Table 2, an average caries prevalence of 17, 9% was recorded in the permanent dentition in this study.

**Table 2:** Caries Prevalence in Children with ASD in KwaZulu-Natal (*n* = 149)

Permanent Dentition	Prevalence of Caries (%)	Primary Dentition	Prevalence of Caries n (%)
Molars	51,65	Molars	40,8
Premolars	12,45		
Canines	3,7	Canines	21,88
Incisors	3,7	Incisors	25,45
Average Caries Prevalence	17,9	Average Caries Prevalence	29,38

A mean score for decayed (d), 0, 96; missing (m), 0, 01; and filled (f), 0 was recorded in the primary dentition. The average dmft score recorded was 0, 97. The caries prevalence in the primary molars was recorded at 40, 8%; canines 21, 9%; and incisors 25, 4%. The average caries experience for the primary dentition was 29, 38%.

This study also demonstrated that 16, 8% of the study participants presented with no observable dental caries, whilst 12, 8% presented with at least one decayed tooth as specified in Table 3. The prevalence of decayed teeth observed within gender, location and age is presented in Table 3. These results indicate that males were observed with more dental caries than females. The presence of decay based on location show that more caries was observed in the suburban location. Dental caries within the age category revealed high caries prevalence in the 12–14-year age group.

**Table 3:** Decay Within Gender; Location and Age (*n* = 149)

Category Gender	Decay = 0	Decay = 1	Decay = 2	Decay = 3	Decay = 4	Decay = 5	Decay = 6	Decay = 7	Decay = 8	Decay = 9	Decay = 12	Total
Female	19 (23,3)	5 (11,6)	11 (25,6)	1 (2,3)	7 (16,3)	0 (0)	3 (7,0)	4 (9,3)	0 (0,9)	1 (0)	1 (2,3)	43 (28,9)
Male	15 (14,2)	14 (13,2)	19 (17,9)	7 (6,6)	32 (30,2)	3 (2,8)	9 (8,5)	1 (0,9)	5 (4,7)	1 (0,9)	0 (0)	106 (71,1)
(*p* < 0,05)												
Category Location	Decay = 0	Decay = 1	Decay = 2	Decay = 3	Decay = 4	Decay = 5	Decay = 6	Decay = 7	Decay = 8	Decay = 9	Decay = 12	Total
Peri-Urban	23 (19,8)	14 (12,1)	25 (21,6)	7 (6,0)	24 (20,7)	2 (1,7)	11 (9,5)	4 (3,4)	5 (4,3)	1 (0,9)	0 (0)	116 (77,9)
Rural	2 (6,1)	5 (15,2)	5 (15,2)	1 (3,0)	15 (45,5)	1(3,0)	1 (3,0)	1 (3,0)	0 (0)	1 (3,0)	1 (3,0)	33 (22,1)
(*p* = 0,05)												
Category Age	Decay = 0	Decay = 1	Decay = 2	Decay = 3	Decay = 4	Decay = 5	Decay = 6	Decay = 7	Decay = 8	Decay = 9	Decay = 12	Total
7–8 Years	11 (36,7)	4 (13,3)	8 (26,7)	1 (3,3)	6 (20,0)	0 (0)	0 (0)	0 (0)	0 (0)	0 (0)	0 (0)	30 (20,1)
9–10 Years	5 (13,5)	3 (8,1)	8 (21,6)	3 (8,1)	12 (32,4)	1 (2,7)	2 (5,4)	2 (5,4)	0 (0)	I (2, 7)	0 (0)	37 (24,8)
11–12 Years	5 (13,5)	5 (13,5)	9 (24,3)	2 (5,4)	8 (21,6)	1 (2,7)	4 (10,8)	1 (2,7)	1 (2,7)	0 (0)	1 (2,7)	37 (24,8)
13–14 Years	4 (8,9)	7 (15,6)	5 (11,1)	2 (4,4)	13 28,9)	1 (2,2)	6 (13,3)	2 (4,4)	4 (8,9)	1 (2,2)	0 (0)	45 (30,2)
(*p* = 0,05)												

The assessment of the gingiva indicated that majority of the participants presented with mild gingival inflammation (46, 3%). The results of the PI in the current study as indicated in Table 4 show the following mentionable scores, namely, a film of visible plaque was recorded at 43, 6% and moderate accumulation of plaque at 42, 3%.

**Table 4:** Adapted Gingival Index and Plaque Index [38] of children with Autism spectrum Disorder in KwaZulu- Natal (*n* = 149)

Gingival Status	Average Gingival Index Score n (%)	Plaque Index	Plaque Index n (%)
Healthy Gingiva	6 (4)	No plaque	14 (9,4)
Mild Gingival Inflammation	69 (46,3)	A film of visible plaque after the use of a disclosing agent or probe.	65 (43,6)
Moderate Gingival Inflammation	66 (44,3)	Moderate accumulation of plaque on more than one third of the tooth surface, Can be seen with the naked eye.	63 (42,3)
Severe Gingival Inflammation	8 (5,4)	Accumulation of plaque on more than two thirds of the tooth surface. Presence of calculus.	7 (4,7)
Total	149 (100)	Total	149 (100)

The results of the examination on attrition indicated that 40,9% of the participants showed no signs of dentine wear; 47,0% showed signs of loss of enamel surface characteristics; and 11,4% presented with wearing of the teeth where the dentine was just visible. There were no observations of a dentine exposure of greater than one third. The soft tissue examination indicated that the most prevalent soft tissue trauma was lip biting (37, 25%) Table 5.

**Table 5:** Attrition in Children with Autism spectrum Disorder in KwaZulu-Natal (*n* = 149)

Description of Dentition	Average Attrition Score (%)
No tooth loss (no wear into dentine)	40,9
Loss of enamel surface characteristic	47,0
Tooth loss (with dentine just visible)	11,4
Enamel loss exposing greater than 1/3 of dentine.	0,0
Total	100

## Discussion

To our knowledge this is the first epidemiological study investigating the oral health status of children aged 7 to 14 years with ASD in South Africa. This condition is virtually 5 times more common amongst males than females [40]. According to this ratio, the gender representation in this study was males (71%) and females (29%). Inequality in demographic representation could be attributed to a reduced number of ASD diagnoses in the rural areas [41].

The overall caries prevalence in the permanent dentition of this study population was 85, 2%. This was significantly higher than a similar study conducted in 2011 [2], which reported an overall caries prevalence of 77% in children with ASD in Dubai; the statistics from the National Oral Health survey which indicated a caries prevalence of 41, 7% between 1999 and 2002 [42],; the prevalence of 30, 55% and 56,5% reported in 2014 [29] and 2015 [30], respectively. This is indicative of a greater burden of dental caries for this study population within the South African context. In 1981, the World Health Organization (WHO) and the World Dental Federation (FDI) jointly formulated goals for oral health to be achieved by the year 2000. *“By the year 2000, the global average for dental caries was to be no more than 3 DMFT at 12 years of age.”* [36]This study 17 years later, with an average DMFT of 3, 42 is above the global average, indicating that oral health goals were not met for this study population.

The mean DMFT score recorded in the current study when compared to previous DMFT scores for South African studies shows that it is lower than the mean DMFT recorded in Durban in 1988; same as that recorded in Durban in 2002 [38], but significantly higher than the study in Venda, South Africa [28].When compared to DMFT scores on studies conducted abroad with Children with ASD [2, 9, 24, 28–30, 34], the current DMFT scores are higher, except for the study conducted in 2014 [17]. The DMFT of the current study indicates high caries prevalence in the permanent dentition of children with ASD in KwaZulu-Natal when compared to both South African studies and studies conducted abroad. These results support previous studies that the burden of dental caries in the permanent dentition of children with ASD is of concern [28]. This may be due to the presence of more permanent teeth in the oral cavity as the child’s age increases.

This study also demonstrated that both maxillary and mandibular molars (51, 7%) presented as the major contributors to the decayed component of the DMFT. Previous reports have indicated the preference for soft and sugary foods, further compounded by the pouching of food at the back of the oral cavity, rather than swallowing it, to be accountable for the high prevalence of caries in the molar region of children with ASD [43]. Oral care practices at home and the frequency of brushing have also contributed to caries levels in children with ASD [44]. Increased caries noted in this study population might also be as a result of challenges faced by parents during oral care routines [45], and manual dexterity required for adequate tooth brushing resulting in poor and unsatisfactory plaque control and removal [2].

The dmft recorded for this study is lower than the mean dmft score of the recent study, conducted in KwaZulu-Natal [31]. However important to note is that fact that despite a low dmft index, once again the major contributors to the decay component in the primary dentition was the molars (40, 8%). This further supports findings of the pouching of food at the back of the mouth for extended periods of time as a significant contributing factor to dental caries in this population [41].

The decayed teeth recorded in both the permanent and primary dentition in this study were all untreated, highlighting unmet dental needs amongst children with ASD [46]. The mean filled component for the DMFT/dmft was recorded as, 0 in this study. This supports previously reported studies of unmet dental needs, poor access to restorative dentistry, poor preventive care or an aversion to dental treatment in children with ASD [47]. Dental care access and oral care delivery has proved to be very problematic for children with ASD. Unmet dental needs within the South African context may be as a result of core liabilities such as financial costs and scarcity of medical aid coverage for this population [12].

This study showed that 16, 8% of children with ASD presented with no dental caries and 12, 7% presented with at least one decayed tooth. This can be attributed to families and caregivers monitoring dietary intake, thereby limiting the consumption of cariogenic foods. Moreover, efficient supervision of parents during home oral care practices has contributed to children with ASD having a low or zero caries prevalence [48]. This emphasizes the need for inclusion of family members and caregivers during the planning of oral care programs for children with ASD. The distinctive obsession with certain low carbohydrate diets have also impacted dental caries positively by reducing the risk to dental caries [39].

This study consisted of 106 males and 43 females, revealing a male-female ratio of 2.5:1, which was undeviating from former a study reporting a male gender bias in ASD [49]. A Chi square test was performed to determine whether there was a statistically significant relationship between the prevalence of dental caries and gender. The *p*-value between *“gender”* and *“dental caries”* is 0.049 (*p* < 0.05). Significantly more males exhibited dental caries than females. This finding was contradictory to findings that demonstrated that females exhibited higher caries prevalence than males [20, 41]. These results were however consistent with findings in a study in Chennai that indicated higher prevalence of caries in males with ASD [35].

Females clinically diagnosed with ASD are most likely to be at the advanced end of the IQ spectrum [50, 51]_._ Furthermore, females with ASD that have an “average” IQ demonstrate amplified functional attributes compared to their male counterparts [52]. This may account for better oral care practices amongst females, thereby exhibiting better oral hygiene and fewer caries. The results of caries prevalence within gender in this study may also be attributed to the sample size.

The *p*-value between *“age”* and *“dental caries”*, (*p* = 0.05), indicated a significant relationship between *“age”* and *“dental caries”*. The prevalence of caries recorded in this study was predominantly in the 12 to 14 year age category (30, 2%). This finding is in line with the findings of an almost doubled DMFT score for the 12 to 15 year age in Venda, South Africa [28]. The results of this study are also similar to the report showing that the 12 to 15 year age group was in great need of preventative and restorative dental services in South Africa [33]. An increase in age is accompanied by an increase in the number of permanent teeth present in the oral cavity. The finding of an increased prevalence of caries in this age group may be attributed to the increased number of permanent teeth present. Preventative oral health programs targeted at this specific age group should be tailored to include fissure sealants on all newly erupted molars. Fissure sealants have been demonstrated as an efficient dental caries preventative measure [53]. Assistance provided by family members during oral care practices also tend to become more challenging as the child ages and this has impacted the oral care status and the prevalence of dental caries [18]. Besides this being the age during which permanent molars replace deciduous premolars, it is also the age at which adolescence emerges and the child tends to declare independence requiring less family assistance and supervision during oral care routines. Oral care program should also include practices to assist this newly found independence in this specific age group.

An investigation into the prevalence of dental caries and geographical location indicated that participants located in the peri-urban area presented with a higher prevalence of dental caries than those in the rural areas. Similar results were obtained in 2014 [28]. Due to affordability and socioeconomic implication, access to sugar and sugar containing foods are limited in rural areas, accounting for the low caries prevalence in these areas [54].

The data analysis indicates an overall prevalence of gingival inflammation of 94%. A high prevalence of mild gingival inflammation was recorded (46, 3%). Some of the challenges for optimal gingival status include behaviors in the dental office and at home, oral care practices; insufficient manual dexterity and deficient oral hygiene knowledge [2, 36]. Research has indicated that reduced upper limb fine motor coordination tends to produce challenges for the maintenance of optimum oral hygiene in children with ASD, thus increasing the probability of gingival inflammation [18]. Furthermore the frequently consumed psychotropic medication has also impacted the gingival status in ASD due to its side effects such as gingival hyperplasia [55].

The plaque index scores indicate that most of the children presented with either a film of visible plaque or moderate plaque accumulation. This finding is in agreement with previous findings [2, 13, 16, 17], which recorded poor oral hygiene in children with ASD as result of plaque accumulation. Rearing a child with ASD is an overwhelming experience for all involved, frequently resulting in stressful situations. In an attempt to minimize these stressful situations, adaptations are made to the child’s routines. This leads to interferences in the oral health care regimen [56]. Children with ASD often do not practice proper oral care routines due to an aversion to the presence of a toothbrush in their mouth and a dislike of the taste and texture of toothpaste. Consequently they tend to brush their teeth less frequently than children without ASD [36].

Attrition scores (47%) in the mild category were noteworthy in this study. Bruxism, a form of vigorous grinding of teeth has being reported as one of the sleep problems frequently experienced by children with ASD [57]. Reports indicate a higher prevalence of bruxism in children with special needs resulting in extreme dental wear, avulsion of teeth and temporomandibular joint pain [16].

Soft tissue trauma in the head and neck region was found in 68, 5% of the study participants. This is in agreement with past reports that almost 70% of autistic individuals present with self-injurious behaviours located in the head and neck region [9]. Of the 68, 5% that presented with soft tissue trauma, lip biting (37, 25%) was the most prevalent. The high prevalence of lip biting in this study may also be due to the distorted pain tolerance that has been noted in children with ASD [21].

### Clinical relevance

Optimum oral health is an essential component to the overall maintenance of optimum general health for children with ASD. This vulnerable special needs population has been posited as *“at risk”* for the development of non-communicable dental diseases such as dental caries, a condition that is very prevalent amongst these children. The expansive knowledge of dental caries and the preventative measures available, renders the effective prevention of dental caries a reasonably manageable objective. Research findings will henceforth serve to inform the development and implementation of applicable and efficient oral health preventative programs. These programs will not only serve to address identified areas in the research findings but also attempt to moderate the access to care and minimize oral health care inequalities as identified in unmet dental needs in this study population. The findings of this study will also serve as an important data source during the creating, assimilating, evolving, and implementing of community preventative oral care programs for children with ASD.

## Conclusion

The results of this study indicate that the children aged between 7 to 14 years with ASD in KwaZulu-Natal, displayed an average DMFT of 3, 42. The molars presented as significant contributors to the DMFT/dmft recorded in this study. Furthermore all of the carious lesions recorded were untreated. There was no evidence of any restorative or preventative treatment measures. Unmet dental needs are therefore an important concern in this population. Mild gingival inflammation and evidence of the presence of moderate plaque accumulation further emphasize the need for preventive intervention in this study population. Health care planners should develop preventive programs that are targeted at caries prevention; oral care practices and oral care education to address the various oral care challenges in ASD.

## Abbreviations

ASD: Autism spectrum disorder
DOE: Department of Education
DOH: Department of Health
IBM SPSS: SPSS Statistics is a software package used for logical batched and non-batched statistical analysis. Long produced by SPSS Inc., it was acquired by IBM in 2009. The current versions are officially named IBM SPSS Statistics
KZN: KwaZulu-Natal
SIB: Self-injurious behaviors
WHO: World Health Organization

## Footnotes

1 BE036/16

2 Ref: 2/4/8/770

3 NHRD Ref: KZ_2015RP54_93
